# Functional status analysis of RNH1 in bladder cancer for predicting immunotherapy response

**DOI:** 10.1038/s41598-023-39827-7

**Published:** 2023-08-03

**Authors:** Sen Chen, Jun Ran, Zhouqian Fan, Mingyou Liu, Liang Wu, Qiude Li, Jian Peng, Zuquan Hu

**Affiliations:** 1 Immune Cells and Antibody Engineering Research Center in University of Guizhou Province, Key Laboratory of Biology and Medical Engineering, School of Biology and Engineering (School of Modern Industry for Health and Medicine), Guizhou Medical University, Guiyang, 550025 China; 2 Key Laboratory of Environmental Pollution Monitoring and Disease Control, Ministry of Education, Guizhou Medical University, Guiyang, 550025 China

**Keywords:** Cancer therapy, Tumour biomarkers, Tumour immunology, Cancer

## Abstract

Bladder cancer (BLCA) typically has a poor prognosis due to high rates of relapse and metastasis. Although the emergence of immunotherapy brings hope for patients with BLCA, not all patients will benefit from it. Identifying some markers to predict treatment response is particularly important. Here, we aimed to determine the clinical value of the ribonuclease/angiogenin inhibitor 1 (RNH1) in BLCA therapy based on functional status analysis. First, we found that RNH1 is aberrantly expressed in multiple cancers but is associated with prognosis in only a few types of cancer. Next, we determined that low RNH1 expression was significantly associated with enhanced invasion and metastasis of BLCA by assessing the relationship between RNH1 and 17 functional states. Moreover, we identified 95 hub genes associated with invasion and metastasis among RNH1-related genes. Enrichment analysis revealed that these hub genes were also significantly linked with immune activation. Consistently, BLCA can be divided into two molecular subtypes based on these hub genes, and the differentially expressed genes between the two subtypes are also significantly enriched in immune-related pathways. This indicates that the expression of RNH1 is also related to the tumour immune response. Subsequently, we confirmed that RNH1 shapes an inflammatory tumour microenvironment (TME), promotes activation of the immune response cycle steps, and has the potential to predict the immune checkpoint blockade (ICB) treatment response. Finally, we demonstrated that high RNH1 expression was significantly associated with multiple therapeutic signalling pathways and drug targets in BLCA. In conclusion, our study revealed that RNH1 could provide new insights into the invasion of BLCA and predict the immunotherapy response in patients with BLCA.

## Introduction

Bladder cancer (BLCA) is one of the most common malignancies of the urinary system^1^. BLCA can be classified according to pathological and clinical manifestations as non-muscle invasive BLCA (NMIBC) and muscle invasive BLCA (MIBC)^2^. Transurethral resection of the bladder tumour (TURBT) is the mainstay therapy of those with NMIBC^3^, but they almost always recur and may progress to invasive BLCA. Radical cystectomy is the standard therapy for patients with MIBC^4^. However, because MIBC can rapidly metastasize to lymph nodes, liver, lungs, bone, and brain, approximately 50% of patients eventually develop distant disease due to disseminated micrometastases^5–7^. Although the combinations of radiotherapy, immunotherapy, and chemotherapy have improved survival^8,9^, most patients still experience relapse and metastasis, which leads to a poor prognosis. An important reason is the high functional heterogeneity of cancer cells. This underscores the urgent need to develop a method to decode the functional state of cancer cells and to identify some markers that affect specific functional states for predicting treatment response. In addition, it is vital to find a key molecule that can remodel the noninflamed tumour microenvironment (TME) into an inflamed TME and has the potential to be a combination therapy target^10^.

Ribonuclease/angiogenin inhibitor 1 (RNH1), a ribonuclease inhibitor (RI), is a ubiquitously expressed protein in the cytoplasm, nucleus, mitochondria as well as on endoplasmic reticulum^11^. RNH1 interacts with angiopoietin (ANG) to inhibit its ribonuclease activity, and can also bind to the 40S ribosome and control translation^12^. For example, RNH1 binds to ribosomes and regulates erythropoiesis by controlling specific translation of the erythroid transcription factor GATA1^13^. Additionally, several studies have identified that RNH1 is involved in various functional states such as cell differentiation, proliferation, invasion, migration, apoptosis, autophagy, and the cell cycle, apart from its ribonuclease inhibitor function. Rnh1 promotes differentiation and myelination via RhoA in oligodendrocytes in *Rattus norvegicus*^14^. RNH1 is essential for adult haematopoietic stem cell function and cell cycle regulation^15^. In cancer, RNH1 inhibits the proliferation of mouse melanoma^16^; overexpression of RNH1 in colorectal cancer cells HT29 resulted in increased autophagy^17^. Studies have also found that the upregulation of RNH1 inhibits cell proliferation, migration and invasion in BLCA, alters cell morphology and adhesion and leads to rearrangement of the cytoskeleton in vitro^18^. However, the application value of RNH1 in BLCA therapy has not been described and its role in immunotherapy is unclear.

In the present study, RNH1 was found to be abnormally expressed in various cancers based on pancancer analysis. Based on the CRDscore algorithm developed by He et al.^19^, we developed the R package *FUNscore* to assess tumour functional status and revealed that low expression of RNH1 was significantly associated with enhanced invasion in BLCA. In addition, this invasive effect is associated with immune activation and molecular subtypes of BLCA. Importantly, RNH1 can serve as a marker of invasion and has the potential to predict the therapeutic response to immunotherapy in BLCA.

## Materials and methods

### Data retrieval and download

The Cancer Genome Atlas (TCGA) data: the HTSeq–Counts data and the corresponding survival and clinicopathological information of cancer patients were downloaded from the TCGA data portal using the software package *TCGAbiolinks* (v2.20.0)^20^ in R (https://www.r-project.org/, v4.1.0) (accessed on 1 August 2021). For cancer types with no normal samples, or with less than ten normal sample number, we downloaded standardized data from the UCSC Xena data portal integrating the Genotype-Tissue Expression (GTEx) dataset. The abbreviations for various cancer types are given in Table S1.

Gene Expression Omnibus (GEO): Six BLCA GEO cohort datasets, namely GSE31684, GSE5287, GSE48057, GSE48277, GSE13507, and GSE69795, were downloaded using the software package *GEOquery* (v2.64.2) in R. Detailed information on these datasets is available in Table S2.

Based on the Creative Commons 3.0 Licence, the complete expression data and detailed clinical information of a BLCA immunotherapy related IMvigor210 cohort (patients treated with atezolizumab) were obtained from the R data package *IMvigor210*^21^.

### Expression, genetic alteration and methylation analysis of RNH1

The expression of the RNH1 gene in normal and tumour tissues was analysed in R software. To assess the expression of RNH1 protein in different cancers, we used data from Clinical Proteomic Tumor Analysis Consortium (CPTAC) in the UALCAN web service for analysis^22,23^. We also used UALCAN to analyze the promoter methylation level of RNH1. We utilized the cBioPortal web service^24,25^ for analysis the genetic alteration status of RNH1 based on the TCGA cohorts.

### Survival and prognosis analysis

For the prognostic analysis of RNH1 expression in across cancers, we used the GEPIA2 tool to perform overall survival (OS) and relapse-free survival (RFS) analyses. In the ‘Survival Analysis’ module on the GEPIA2 web server^26^, samples were stratified into high and low expression groups according to the median expression of the RNH1 gene in each cancer type, and OS and RFS analyses were performed. Similarly, we used cBioPortal to analyse the correlation between the genetic alteration status of RNH1 and disease-free survival (DFS) across cancers^27^. In addition, we used the *survival* package in R software to perform OS analysis for other specific groups.

### Calculation of functional status score

We downloaded 14 functional gene signatures (FUNGs) from the cancerSEA database^28^. Additionally, the collection of autophagy-related and ferroptosis-related genes was obtained from the HADb^29^ and FerrDb^30^ databases, respectively. A study identified gene signatures associated with tumour immune escape in mice^31^, and we converted these gene signatures to human orthologues. A total of 17 collected FUNGs (Table S3) were used to assess the functional status score (${FUN}_{score}$).

He et al.^19^ developed an algorithm that accounted for variation in the signal‐to‐noise ratio across genes and cells based on the expression profiles of circadian‐related genes to assess the level of circadian rhythm disruption. Here, we extend the algorithm to a broader field for assessing the functional status of tumour samples or cells. The specific steps were as follows:

First, for a given normalized expression matrix *C* of bulk RNAseq, microarray, or TPM for scRNA‐seq, we define the average expression *E*_*i*_ of gene *i* across *N* samples or cells as:

$${E}_{i}=\left\{\begin{array}{c}\frac{\sum_{j}{C}_{i,j}}{N}, j=\mathrm{1,2},3,\dots ,N;bluk\,RNAseq\,or\,microarray\,data \\ {log}_{2}\left(\frac{\sum_{j}10 \times \left({2}^{{C}_{i,j}}-1\right)}{N} +1\right), j=\mathrm{1,2},3,\dots ,N;scRNAseq\end{array}\right.$$

Next, a random sampling strategy was employed. All genes were divided into 50 expression bins according to their average expression *E*_*i*_, and the frequency of FUNGs in each bin was counted and designated *B*_*FUNGs*_. The signature genes *K* were randomly selected with the same number of FUNGs in each bin and iterated 1000 times. Subsequently, we define a centred gene expression matrix that can be interpreted as the data without an excessive migration signal:

$${X}_{i,j}= {C}_{i,j} - \frac{\sum_{j}{C}_{i,j}}{N}, j=\mathrm{1,2},3,\dots , N,$$

where *X*_*i,j*_ represents the central expression of gene *i* in the sample or cell *j*.

Finally, a random score, ${S}_{random}$, as the mean of *K* × 1000 random signatures sampled above and the FUNGs score, ${S}_{FUNGs}$, as the mean of K FUNGs using the centred expression data of each sample or cell were used to quantify the abundance of FUNGs. The ${FUN}_{score}$ which was normal or a mixture of normal distributions, was calculated as follows:

$${FUN}_{score}= {S}_{random} - {S}_{FUNGs}$$

A cut-off of 75% based on quartiles was used as the threshold for ${FUN}_{score}$ in single cells, while the median was used for bulk RNAseq. This tool was implemented as an R package that is documented and freely available at https://github.com/BioInfoNote/FUNscore.

### Weighted gene coexpression network analysis (WGCNA)

First, we obtained an expression data profile containing only genes encoding proteins significantly correlated with RNH1 expression (|R|> 0.2 and *p* value < 0.01, Table S4). The *WGCNA* package^32^in *R* software was employed to execute the WGCNA analysis. In the process, we set the minimum module size (minClusterSize = 30) and soft-thresholding power (power = 6) for network construction. Subsequently, we calculated the correlation of each module with 17 ${FUN}_{score}$. In WGCNA, GS is defined as the correlation between a gene and a phenotype, and MM is defined as the measure of the importance of a gene in a module according to the formula MM(i) = cor (x_i_, ME). In this study, a gene with GS > 0.6 and MM > 0.8 was defined as a hub gene among the candidate gene modules.

### Differential gene expression and gene enrichment analysis

Differential expression analysis of RNA was performed using the DESeq2^33^ package in R. Genes with the parameters of FDR < 0.01 and |fold change|≥ 1.5 were considered differentially expressed genes (DEGs). Gene Ontology (GO) enrichment analysis was performed using the *clusterProfiler* package (v4.0.2)^34^ in R.

### Molecular subtypes and consensus clustering of BLCA

We used the R package consensusMIBC^2^ to infer the consensus subtype of BLCA, which included a combined consensus subtype and six published molecular classifications (University of North Carolina (UNC), Baylor, Cancer Genome Atlas (TCGA), MD Anderson Cancer Center (MDA), Lund and Cartes d’Identité des Tumeurs (CIT)). Additionally, we inferred consensus clusters based on the 95 hub gene signatures using the R package *ConsensusClusterPlus*^35^. The optimal cluster number k was chosen depending on the elbow and CDF curve. Principal component analysis (PCA) was performed to evaluate the difference between the clusters. Molecular subtype information is available in Table S5.

### Calculation of the T-cell inflammation score

Ayers et al.^36^ developed and validated a pancancer T-cell-inflamed score, which could define preexisting cancer immunity, and predict the clinical response of immune checkpoint blockade (ICB) therapy. Here, we used a model to calculate the T-cell inflammation score. The calculation formula was as follows:

$$\mathrm{Score}=\sum {{\beta }_{i} \times E}_{i} ,$$

where ${\beta }_{i}$ is the coefficient of gene i obtained from the above model, ${E}_{i}$ is the expression of gene i, and information on genes and coefficients is available in Table S6.

### Immune cell infiltration analysis

Single-sample gene set enrichment analysis (ssGSEA)^37–39^ was used to evaluate the fractions of 28 tumour-infiltrating immune cell (TIIC) phenotypes in the tumour microenvironment. To avoid computational errors caused by a single algorithm and different sets of marker genes for TILs, we downloaded immune infiltrate data evaluated using the CIBERSORT^40^, CIBERSORT-ABS^41^, EPIC^42^, MCP-counter^43^, quantTIseq^44^, xCell^45^, TIMER^46^ and TIDE algorithms for the 33 cancer types from the TCGA database using the TIMER2 web server^47^.

### Enrichment analysis of various therapeutic signatures

Gene signatures of several potential therapeutic pathways were collected from the study by Hu et al.^48^ (Table S7). Twelve BLCA signatures that are specific to different molecular subtypes were collected from the study performed by the Bladder Cancer Molecular Taxonomy Group^2^ (Table S8). ssGSEA was employed to calculate the ssGSEA score according these gene signatures. The immunotherapy response data for the IMvigor210 cohort were used to evaluate RNH1 expression in the PR/CR group. Additionally, we further used the data of BLCA-related drug target genes (Table S9) obtained from the DrugBank database (https://go.drugbank.com/) to compare their expression in RNH1 groups and BLCA subtypes.

### Statistical analysis

Correlations between variables were explored using Spearman or Pearson coefficients. Continuous variables that conformed to the normal distribution were compared using independent t-tests for comparisons between binary groups, while continuous variables with skewed distributions were compared with the Mann–Whitney U test. Survival curves for categorical variable prognostic analyses were generated using the Kaplan–Meier method, while the log-rank test was used to estimate statistical significance. The significance level was set at *p* < 0.05, and all statistical tests were two-sided. All statistical data analyses were performed using the *R* software or online analysis tools described in the relevant Materials and Methods subsections.

### Ethical approval

Our study did not require an ethical board approval because this article does not contain any studies with human participants or animals performed by any of the authors.

## Results

### Pancancer expression pattern and prognostic significance of RNH1

**Figure 1:**
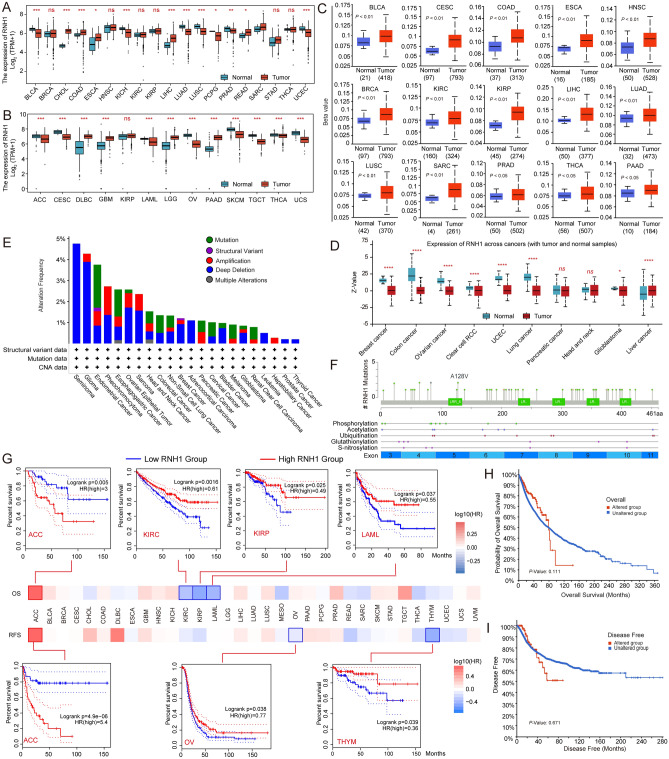
Pancancer expression pattern and prognostic significance of RNH1. (**A**) *RNH1* expression levels in 19 cancer types based on the TCGA database. (**B**) *RNH1* expression levels in 13 cancer types based on the TCGA and GTEx database. (**C**) The promoter methylation level of *RNH1* in 15 cancer types based on TCGA cohorts of UALCAN database. (**D**) Total RNH1 protein expression levels in normal and primary tumour based on the CPTAC dataset from UALCAN. (**E**) The frequency of different *RNH1* genetic alterations in different tumour types based on TCGA cohorts of cBioPortal. (**F**) Statistics associated with RNH1 mutation sites in different tumour types based on TCGA cohorts of cBioPortal. (**G**) Correlation between *RNH1* gene expression and overall survival/disease-free survival in different tumour types in TCGA, assessed using GEPIA2. (**H**–**I**) Potential correlation between *RNH1* alteration status and overall, and disease-free survival in pancancer, as analysed using the cBioPortal tool. *ns* no significant difference; *p < 0.05; **p < 0.01; ***p < 0.001; ****p < 0.0001.

First, we explored the expression of RNH1 in human cancers based on the TCGA, GTEx, and CPTAC databases. We found that the mRNA expression level of RNH1 was low in tissues of 15 cancer types (BLCA, KICH, LUAD, LUSC, PCPG, PRAD, UCEC, ACC, CESC, LAML, OV, SKCM, TGCT, THCA, and UCS) compared with normal tissues (Fig. 1A,B). In contrast, we observed that RNH1 mRNA was highly expressed in tissues of 11 cancer types (CHOL, COAD, ESCA, LIHC, READ, SARC, DLBC, GBM, LGG, PAAD, and SKCM) (Fig. 1A,B). In addition, DNA methylation is an important factor in the regulation of gene expression. Therefore, we explored the promoter methylation level of RNH1. We found a significant increase in the promoter methylation level of RNH1 in tissues of 15 cancer types (BLCA, BRCA, CESC, COAD, ESCA, HNSC, KIRC, KIRP, LUAD, LUSC, SARC, PRAD, THCA, PAAD, and THCA) compared to normal tissues (Fig. 1C). This may be related to the low RNH1 expression in multiple cancer types. Consistently, RNH1 protein levels were low in tissues of multiple cancer types compared with healthy tissue (Fig. 1D).

Next, we used the cBioPortal tool to analyse the genetic alteration status of RNH1 in different cancer types from the TCGA cohorts. We observed the highest RNH1 alteration frequency among patients with seminoma, followed by glioma (Fig. 1E). Notably, the RNH1 alterations in both seminoma and adrenocortical carcinoma were all identified as ‘deep deletion’ (Fig. 1E). 'Deep deletion' is the predominant alteration type in most cancers. Additionally, we found that the frequency of arginine to valine mutations at position 128 in the RNH1 protein was the highest among all mutations (Fig. 1F). Moreover, mutations occurred in almost all exons (Fig. 1F).

The aberrant expression pattern of RNH1 in human cancers prompted us to explore its prognostic value. Therefore, we performed a pancancer survival analysis concerning OS and RFS. We found that RNH1 expression was significantly associated with OS or RFS in several cancer types. As shown in Fig. 1G, high RNH1 expression was associated with good OS in patients with KIRC, KIRP and LAML, and with good RFS in patients with OV and THYM. Conversely, high RNH1 expression was associated with poor RFS and OS in patients with ACC (Fig. 1G). Similarly, we also used the cBioPortal analysis tool to determine the relationship between prognosis and RNH1 genetic alteration status across cancers. Regrettably, we discovered no significant correlation between altered RNH1 and OS, or DFS (Fig. 1H,I).

Taken together, we determined that RNH1 is aberrantly expressed in multiple cancers but is associated with prognosis in only a few types of cancer. The relationship between this aberrant expression and human cancers requires further exploration.

### RNH1 affects multiple functional states in BLCA

**Figure 2:**
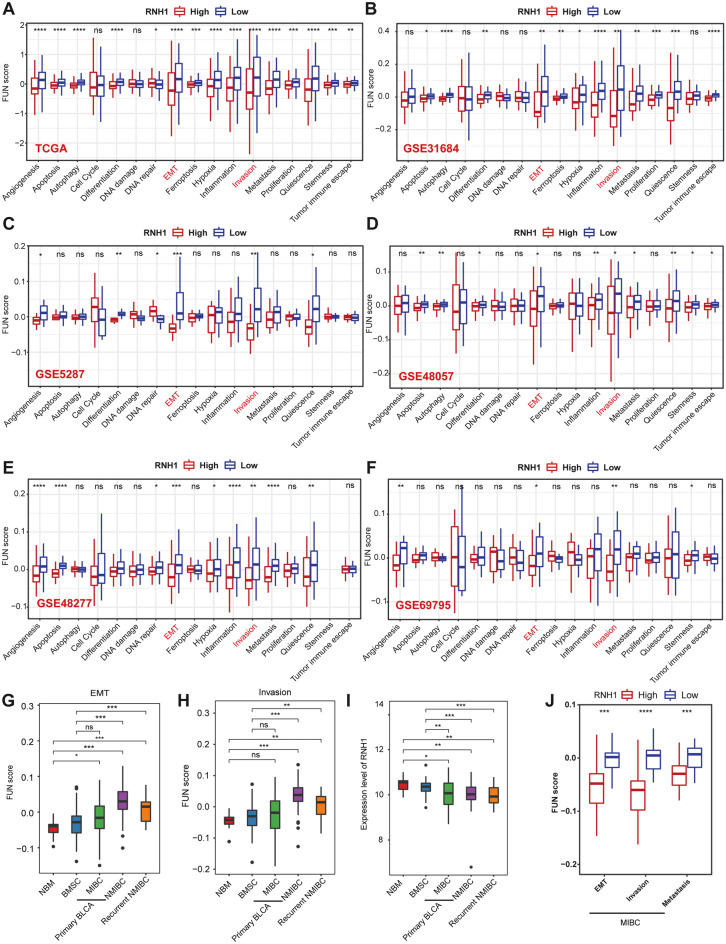
RNH1 affects functional states in BLCA. (**A**–**F**) The differences in FUN scores between groups with high and low *RNH1* expression based on TCGA (**A**), GSE31684 (**B**), GSE5287(**C**), GSE48057 (**D**), GSE48277 (**E**), and GSE69795 (**F**) datasets. (**G**–**I**)The expression of RNH1 (**G**), and the FUN score of both EMT (**H**) and invasion (**I**) were evaluated in different tissues based on GSE13507 datasets. (**J**) The differences in FUN scores between groups with high and low *RNH1* expression based on the GSE13507 dataset. *ns* no significant difference; *p < 0.05; **p < 0.01; ***p < 0.001; ****p < 0.0001.

Pancancer analyses were performed to explore whether there are commonalities in the function of RNH1 in human cancers and to explore its functional role. Therefore, we evaluated the 17 functional status scores for each tumour sample. We found that the effect of RNH1 on the functional status of most cancers was not significant (Fig. S1). Notably, RNH1 exhibited a significant association with multiple functional states in BLCA and LIHC (Fig. S1). Fifteen functional states were affected by the expression of RNH1 in BLCA (Fig. 2A). To avoid possible misinterpretation because of confounding factors in the single dataset analysis, we performed the same evaluation on additional independent GEO datasets of BLCA. Although there was a shift in the evaluation results of different datasets, it is possible to be certain that EMT and invasion have coincident results in these BLCA datasets (Fig. 2B–F). The FUN score for EMT and invasion was lower in the high RNH1 expression group (Fig. 2B–F). In addition, we also observed a higher FUN score for both invasion and EMT in BLCA compared to normal bladder mucosae (NBM) or bladder mucosae surrounding cancer (BMSC) (Fig. 2G,H). Consistently, we found that RNH1 was downregulated in BLCA compared to NBM or BMSC (Fig. 2I). This evidence suggested that high RNH1 expression was not conducive to the migration and invasion of BLCA. Indeed, high expression of RNH1 in MIBC was associated with lower FUN score for EMT, invasion, and metastasis (Fig. 2J).

### Identification of hub genes affecting functional status

**Figure 3:**
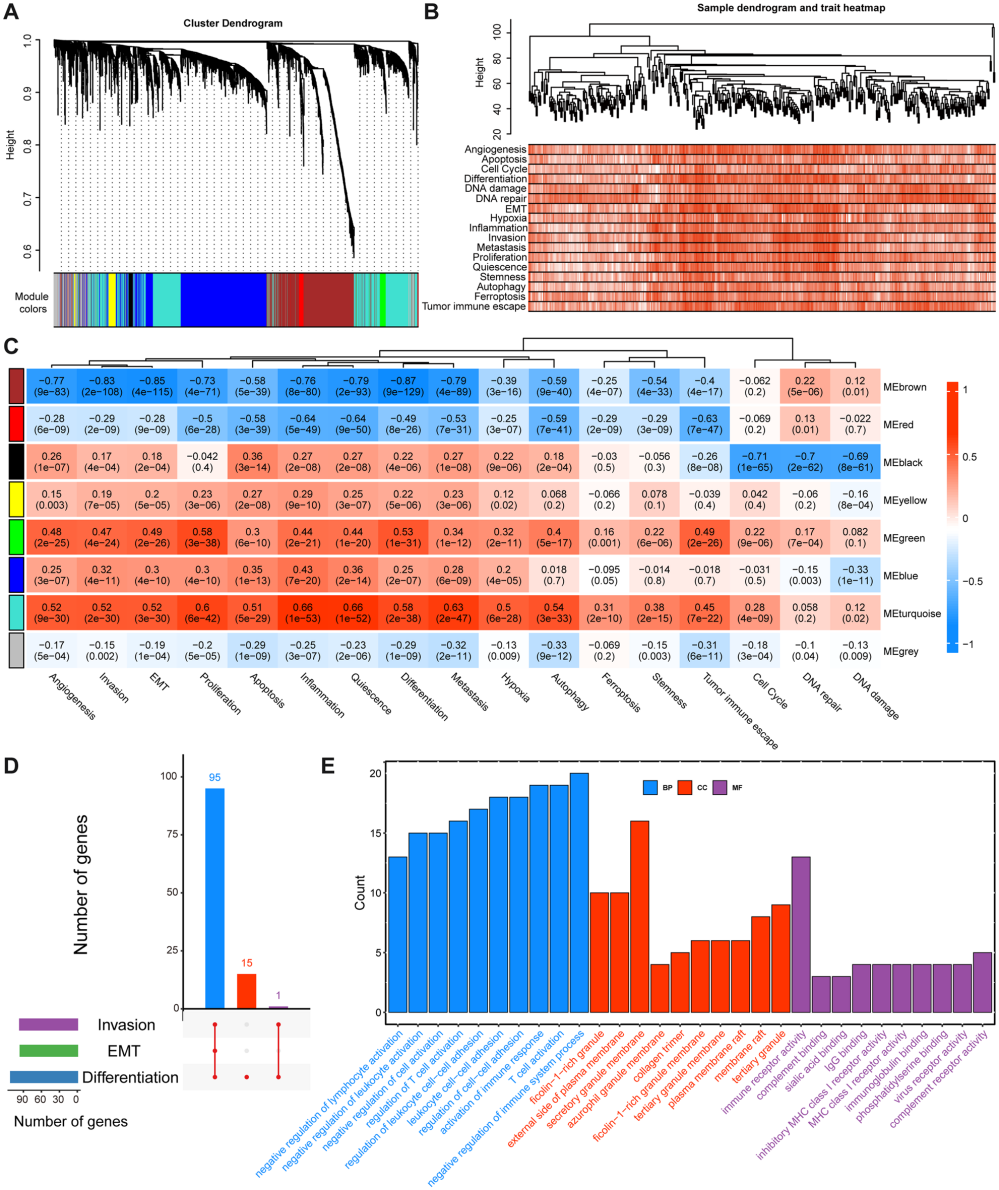
Identification of hub genes affecting functional status. (**A**) The clustering dendrogram of the WGCNA. (**B**) Sample dendrogram and 17 functional status trait heatmap. (**C**) Module-trait relationships. Each row represents a colour module and every column represents a functional status trait. Each cell contains the corresponding correlation and *p* value. Red represents a positive correlation and blue represents a negative correlation. (**D**) UpSet plot of overlapping hub genes of EMT, invasion, and differentiation traits. (**E**) For the overlapping 95 hub genes in the diagram (**D**), the associated BP, CC, and MF were investigated using GO enrichment analysis.

To further uncover the most representative and correlated hub genes affecting functional status, we performed WGCNA based on an expression profile containing only 3416 genes encoding proteins significantly correlated with RNH1 expression to find modules of genes highly correlated with functional status. Eight gene modules were obtained using the dynamic tree cut algorithm, and the RNH1-related genes were determined to predominantly link the brown, turquoise, and blue modules (Fig. 3A). Subsequently, a sample dendrogram and 17 functional statuses trait heatmap were constructed (Fig. 3B), and the correlation between each module and FUNscore of 17 functional status was assessed. We note that only the brown module is significantly linked to multiple functional states, including EMT, invasion, and differentiation (Fig. 3C). This is consistent with our determination above that RNH1 is associated with invasion and metastasis of BLCA. Next, we obtained 95, 96, and 111 hub genes from the brown module that were significantly associated with EMT, invasion, and differentiation, respectively (Table S10). Interestingly, 95 of these genes were all significantly associated with these functional states (Fig. 3D). Finally, we identified significant BP, CC, and MF ontology terms for these 95 genes. For the BP category of the GO analysis, immune response-related processes, such as negative regulation of immune system process, T cell activation, and activation of immune response, were mainly enriched (Fig. 3E). For the CC category, secretory granule membrane, external side of plasma membrane, and ficolin-1-rich granule were significantly enriched, whereas immune receptor activity accounted for the most abundant groups in the MF category (Fig. 3E). This result implied that RNH1 might be associated with immune activation. Moreover, it suggested that this immune activation effect was closely related to EMT and invasion in BLCA.

### RNH1 predicts molecular subtypes of BLCA

**Figure 4:**
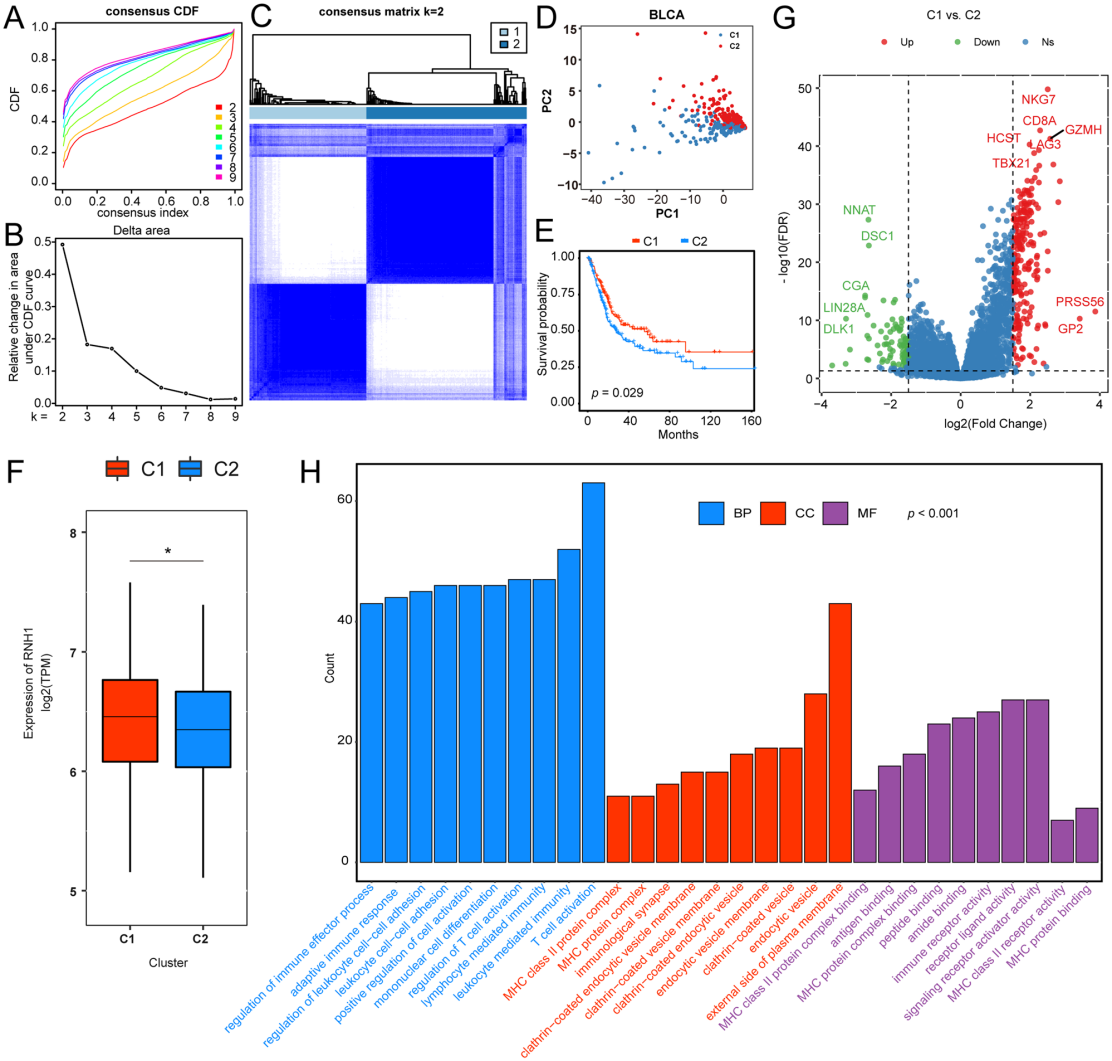
Unsupervised clustering of BLCA using 95 hub gene expression profiles. (**A**,**B**) Consensus CDF curve (**A**) and area (**B**) under the CDF curve when k = 2–9. (**C**) Consensus clustering analysis of TCGA-BLCA samples when k = 2. (**D**) TCGA- BLCA populations identified after unsupervised clustering in panel (**C**). (**E**) Kaplan–Meier survival plot of C1 and C2 groups. A log-rank test was conducted. (**F**) Expression of RNH1 in the C1 and C2 groups. (**G**) Volcano plot of DEGs based on C1 versus C2. (**H**) BP, CC, and MF of DEGs were investigated using GO enrichment analysis.

Molecular subtypes of BLCA have been constructed in several studies. Here, we wondered about the potential of RNH1 for predicting molecular subtypes. ConsensusClusterPlus was used to perform consensus clustering on the samples according to the 95 FUN-related hub genes. The optimal number of clusters was determined based on the CDF. According to the CDF curve and delta area under the CDF curve (Fig. 4A,B), cluster number k = 2 was determined to classify BLCA into two clusters, designated Cluster 1 (C1) and Cluster 2 (C2) (Fig. 4C). Remarkably, principal component analysis separated the two clusters well (Fig. 4D). To assess whether the stratification determined by the FUN-related hub genes was associated with clinical outcomes, we evaluated the prognostic performance of the clusters with respect to OS using Kaplan–Meier survival analysis. Survival analysis showed that C1 had better OS than C2 in the TCGA-BLCA cohorts (Fig. 4E). In addition, we noticed that there was also a significant difference in the expression of RNH1 between the two subtypes, that is, the expression level of RNH1 in the C1 group was higher than that in the C2 group (Fig. 4F). These results suggest differences in expression patterns between the two subtypes. Therefore, we performed differential expression analysis to explore the changes in expression profiles between the two subtypes. A total of 306 DEGs were obtained based on C1 compared to C2, among which, 223 were upregulated and 83 were downregulated (Fig. 4G). To explore the functional roles of these DEGs in the two subtypes of BLCA, GO enrichment analyses were performed using the *clusterProfiler* package. Figure 4H illustrates the top 10 most significantly enriched GO terms (BP, CC, MF) for these DEGs. There was significant enrichment of immune-related terms, such as T-cell activation, leukocyte mediated immunity and lymphocyte mediated immunity in BP, external side of plasma membrane and endocytic vesicle in CC; and receptor ligand activity, immune receptor activity, and signalling receptor activator activity in MF (Fig. 4H). It has been well demonstrated that immunologic activity alteration is a hallmark of the two subtypes. Taken together, these findings demonstrate that by using FUN-related hub genes as probes, we can identify novel patient subtypes with significant clinical outcomes.

### RNH1 promotes tumour associated immune cell infiltration and activates the cancer-immunity cycle

**Figure 5:**
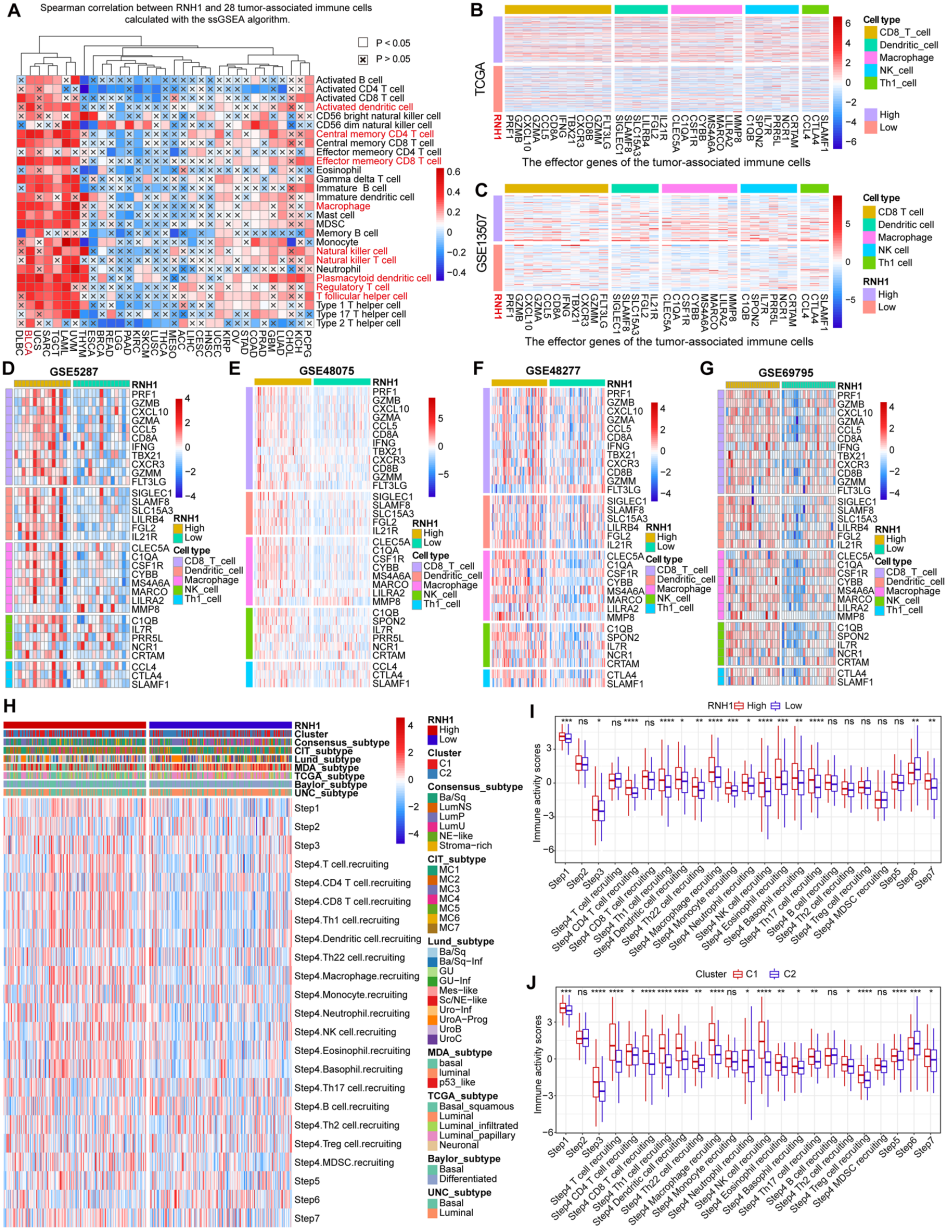
RNH1 promotes the infiltration of multiple immune cells. (**A**) Spearman correlation between RNH1 and 28 TIICs based on RNAseq data of 33 cancer types from TCGA, as calculated using the ssGSEA algorithm. (**B**–**G**) Differences in the effector genes of the above tumour-associated immune cells between high- and low-RNH1 groups from BLCA related datasets: (**B**) TCGA-BLCA, (**C**) GSE13507, (**D**) GSE5287, (**E**) GSE48057, (**F**) GSE48277, and (**G**) GSE69795. (**H**) Differences in various steps of the cancer immunity cycle between different BLCA molecular subtypes. (**I**) Differences in the various steps of the cancer immune cycle between groups with high and low RNH1 expression. (**J**) Differences in the various steps of the cancer immune cycle between groups in C1 and C2.

Based on the above analysis, RNH1 might be associated with the immune response. Next, we further explored the immunological role of RNH1 in BLCA. We found that RNH1 was positively correlated with a majority of immunomodulators in BLCA based on TCGA data, but only a few in other cancer types (Fig. S2). This implies that RNH1 expression in BLCA may influence the TME and play a role in antitumour immunity. Therefore, we estimated the infiltration levels of TIICs based on the ssGSEA algorithm. We discovered a significant positive correlation between RNH1 expression in BLCA and the infiltration levels of 28 TIICs, especially activated dendritic cells, central memory CD4 T cells, natural killer cells, natural killer (NK) T cells, plasmacytoid dendritic cells, regulatory T cells, T follicular helper cell, and macrophages (Fig. 5A). These cells are beneficial for antitumour immune responses. Similarly, RNH1 expression was also significantly positively correlated with the levels of most immune cell infiltrates in several cancer types (USC, SARC, TGCT, LAML, and UVM) (Fig. 5A). Importantly, seven independent immune evaluation algorithms also revealed that the expression of RNH1 in BLCA was positively correlated with the infiltration levels of multiple immune cells such as macrophages (Fig. S3A–G). Consistently, RNH1 was positively correlated with the effector genes of macrophage, CD8 T cells, dendritic cells, and NK T cells (Fig. 5B). This result was confirmed similarly in other independent GEO datasets (Fig. 5C–G). This evidence suggests that BLCA samples with high expression of RNH1 exhibit higher immune cell infiltration. This may contribute to the activation of the immune cell cycle. Therefore, we further evaluated the differences between RNH1 expression and the individual immune activation steps. We noted that samples with high RNH1 expression exhibited higher immune activation scores at multiple immune cycle steps (Fig. 5H,I). In addition, we note that some molecular subtypes are also consistent with the role of RNH1, such as the UNC subtypes (Fig. 5H). Importantly, we found that Group C1 also exhibited higher immune activation scores across multiple immune cycle steps (Fig. 5J). This was consistent with C1 having a better OS than C2. Taken together, these pieces of evidence suggest that RNH1 promotes the infiltration of TIICs and activates the immune cell cycle. In addition, RNH1 may play an important role in immunotherapy.

### RNH1 shapes an inflamed TME and predicts immunotherapy response in BLCA

**Figure 6:**
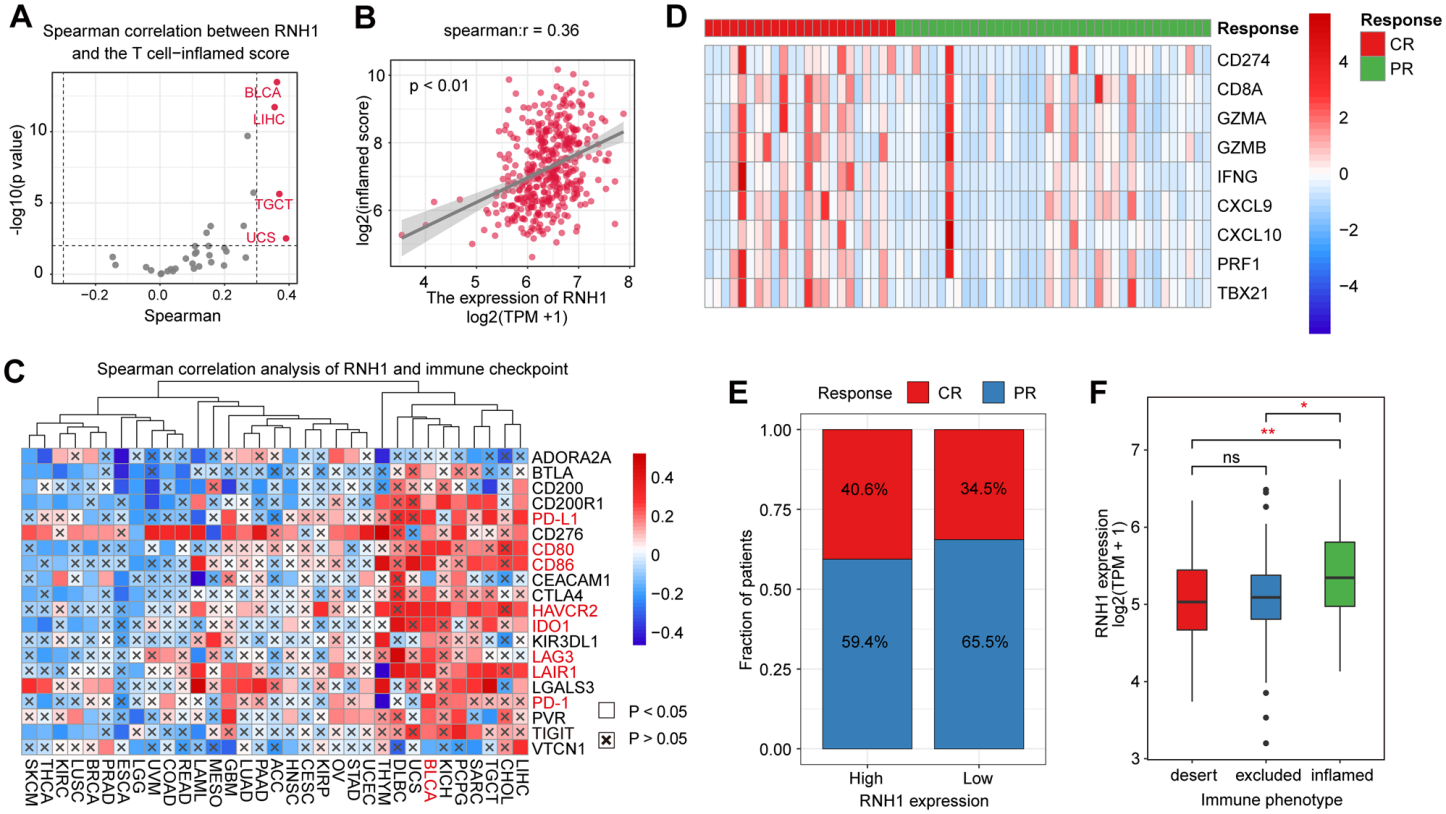
RNH1 predicts the response to immunotherapy in BLCA. (**A**) Spearman correlation analysis of RNH1 and T-cell inflammatory scores in 33 cancer types. (**B**) Scatter plot of Spearman correlation for RNH1 and T-cell inflammation score in BLCA. (**C**) Spearman correlation analysis of RNH1 and 20 typical inhibitory immune checkpoints in 33 cancer types. (**D**) Immune checkpoint expression heatmaps in PR and CR.CR: complete response; PR, partial response. (**E**) Fraction of patients between RNH1 and the clinical response of cancer immunotherapy in the IMvigor210 cohort. (**F**) Expression of RNH1 in inflamed, immune excluded and immune desert tumours from the IMvigor210 cohort. *Ns* no significant difference; * *p* < 0.05; ** *p* < 0.01.

High immune cell infiltration is linked to an immune-inflamed TME^49^. Therefore, we calculated the correlation between the expression of RNH1 and the inflammatory score using a pancancer T-cell inflammation score model developed and validated by Ayers et al.^36^. Consistently, RNH1 expression in BLCA was negatively correlated with T-cell inflammatory scores (Fig. 6A,B). Additionally, ICB therapies have been most effective in patients with expression of immune checkpoints and higher T-cell infiltration^50^. Thus, we further evaluated the correlation between the expression of RNH1 and 20 typical inhibitory immune checkpoints. As expected, RNH1 expression in BLCA was positively correlated with the expression of multiple immune checkpoints, such as PD-L1, CD80, CD86, HAVCR2, IDO1, LAG3, LAIR1, and PD-1 (Fig. 6C). In theory, patients with higher RNH1 expression should have a higher response to ICB because RNH1 defines an inflamed TME. Indeed, the expression of multiple immune checkpoints was higher in patients who had a complete response (CR) to ICB therapy than in patients who had a partial response (PR) in the IMvigor210 cohort (Fig. 6D). Consistently, the proportion of patients responding to ICB treatment in the RNH1 high expression group was higher than that in the low expression group (Fig. 6E). Additionally, the expression level of RNH1 was higher in inflamed tumours than in immune excluded and immune desert tumours (Fig. 6F). Taken together, these results suggest that RNH1 shapes an inflamed TME and has the potential to predict ICB treatment response.

### RNH1 predicts treatment candidates in BLCA

**Figure 7:**
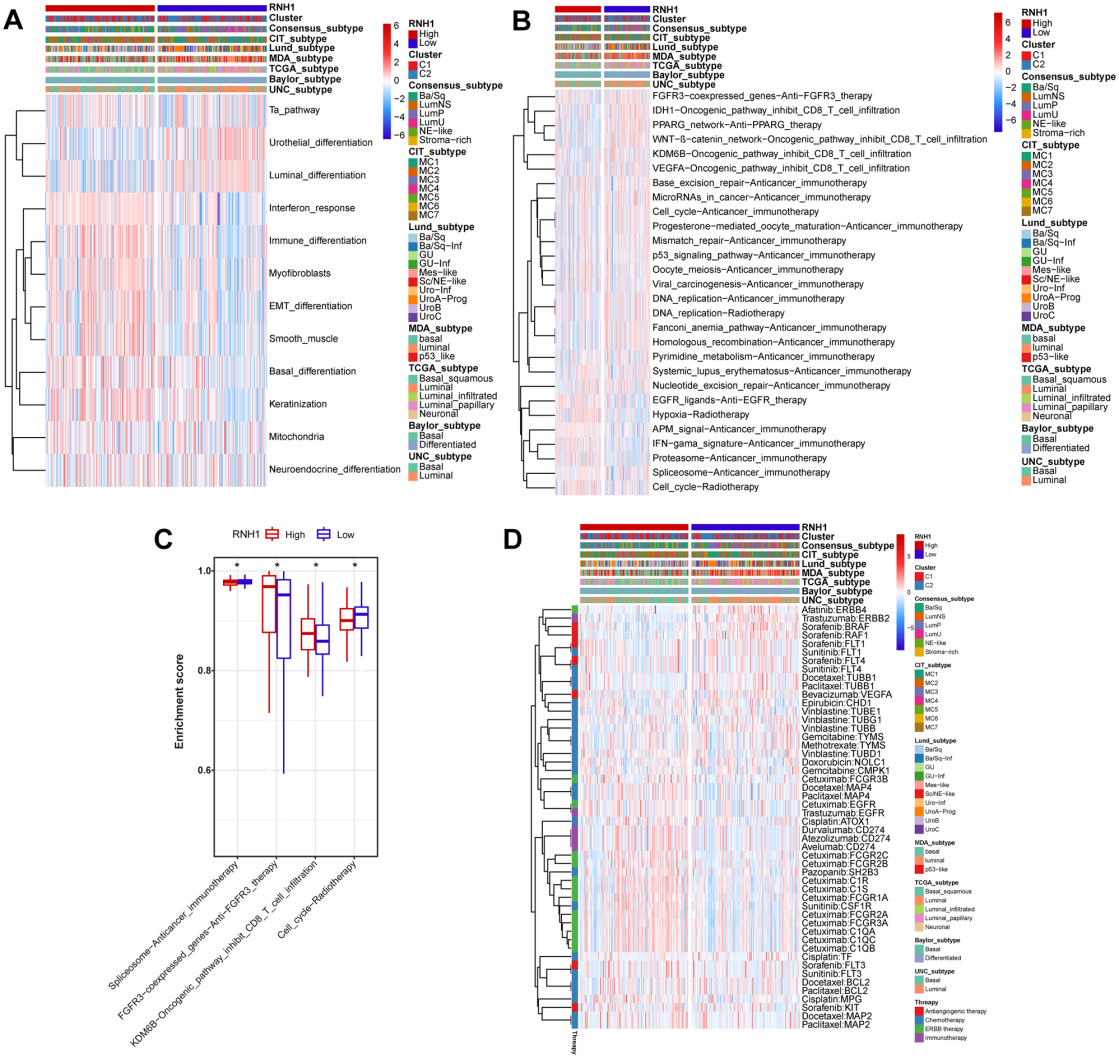
RNH1 predicts the response to several therapies in BLCA. (**A**) Correlations between RNH1 and molecular subtypes using different algorithms and BLCA signatures. (**B**) Correlations between RNH1 and the enrichment scores of several therapeutic signatures based on ssGSEA. (**C**) Boxplot presentation of several therapeutic signatures from panel (**B**). (**D**) Correlation between RNH1 and BLCA-related drug-target genes screened from the DrugBank database. *p < 0.05.

To further explore the potential of RNH1 for predicting treatment candidates in BLCA, we first analysed the relationship between RNH1 expression and different BLCA-related signalling pathways. Surprisingly, changes in RNH1 expression levels significantly affected BLCA-related signalling pathways (Fig. 7A). For example, interferon response, myofibroblasts, smooth muscle, immune differentiation, and EMT differentiation were significantly enriched in the RNH1 high expression group (Fig. 7A). Next, we further explored the relationship between RNH1 expression and various therapeutically relevant signals. We found that RNH1 expression affected certain therapeutic signalling pathways (Fig. 7B), such as the spliceosome, FGFR3, KDM6B, and cell cycle pathways (Fig. 7C). These signalling pathways are involved in immunotherapy, ERBB therapy, chemotherapy, and radiotherapy. Therefore, we collected drug information related to BLCA treatment from the DrugBank database. We then evaluated the relationship between the targets of these drugs and RNH1 expression as well as different subtypes. We found that the targets of multiple drugs showed expression differences between the high and low RNH1 expression groups and different subtypes (Fig. 7D). Significantly, multiple targets of cetuximab were expressed at lower levels in the RNH1 low expression group (Fig. 7D). Taken together, these results show that ICB, chemotherapy, and ERBB therapy can be used, either alone or in combination, for the treatment of BLCA with high RNH1 expression.

## Discussion

BLCA is one of the most common malignant tumours of the urinary system. NMIBC patients often relapse after TURBT, whereas MIBC patients eventually develop distant disease due to disseminated micrometastases. This brings great challenges to the treatment of BLCA, and there is an urgent need to develop more accurate and effective treatment strategies. Identifying effective markers of therapeutic response is a viable strategy. It is generally known that RNH1 plays a role in tumorigenesis by affecting the functional status of tumours in terms of processes, such as cell differentiation, proliferation, invasion, migration, apoptosis, and cell cycle^14–18^. This suggests that multiple important roles of RNH1 in cancer deserve further exploration.

In this study, pancancer analysis indicated that RNH1 was aberrantly expressed in a variety of cancers. Nevertheless, the impact of RNH1 expression on OS and RFS varied depending on the cancer type. It is worth mentioning that our analysis revealed higher methylation levels of RNH1 in multiple cancer tissues than in normal tissues. Although DNA methylation is an important factor affecting mRNA transcription, we found that the methylation level of RNH1 is very low in various cancer tissues. Therefore, the methylation of RNH1 affects its expression, but more experimental evidence is required to support this claim. Additionally, we found that RNH1 exhibits a high rate of deep deletion among genetic alterations in most cancers, but we noted that the total frequency of RNH1 alteration is not high in various cancers. This may be the reason why RNH1 alterations are not associated with OS or DFS.

The high functional heterogeneity of cancer cells presents a major challenge to cancer therapy. In this study, we quantified 17 functional states in tumours based on the CRDscore algorithm developed by He et al.^19^. We found that RNH1 affects multiple functional states in BLCA based on a pancancer analysis. The affected functional status has been demonstrated in several studies. Additionally, we confirmed this finding based on other independent GEO datasets. Importantly, EMT and invasion have coincident results in several GEO datasets. This suggests that RNH1 plays an important role in BLCA by affecting EMT and invasion. Previous studies have shown that metastasis and invasion of bladder malignant tumours are important causes of death in patients^51^. Although studies have found that RNH1 inhibits the migration and invasion of BLCA cells, the underlying molecular mechanism has not been thoroughly studied^18^. Here, we found that RNH1 was downregulated in BLCA compared to NBM or BMSC. In addition, we also observed a higher FUN score for both invasion and EMT in BLCA. More importantly, high expression of RNH1 in MIBC was associated with higher FUN scores for EMT, invasion, and metastasis. These results confirm the reliability of the FUNscore method. This results also suggest that RNH1 may have significant clinical value in BLCA. Consistently, RNH1-related genes were found to be significantly associated with differentiation, invasion, and EMT based on WGCNA. We obtained 95 hub genes related to EMT, invasion, and differentiation. Interestingly, GO enrichment analysis revealed that these genes were significantly enriched in immune activation related pathways. This finding links RNH1 implicated EMT, invasion, and metastasis to immune activation.

In this study, we classified BLCA into two clusters based on 95 hub genes. There was a significant difference in OS between the two clusters. Interestingly, the DEGs derived from the differential expression analysis between the two clusters were also significantly enriched in immune response related pathways. This again confirms that RNH1 mediated EMT and invasion may influence the activation of immune responses. Indeed, based on different algorithms for the assessment of immune cell infiltration, we found a significant positive correlation between RNH1 expression and multiple immune cell infiltrates. In addition, other independent GEO dataset analyses showed that RNH1 expression was significantly correlated with multiple TIICs. In further analysis, we found a significant positive correlation between high RNH1 expression and the expression of multiple immune checkpoints. Meanwhile, RNH1 was significantly positively correlated with the immunotherapeutic response in the BLCA cohort IMvigor210. This is consistent with the most effective outcome of ICB therapy in patients with higher expression of immune checkpoints and T-cell infiltration^50^. Additionally, high immune cell infiltration was linked to an immune inflamed TME^49^. Our analysis also confirms that RNH1 shapes an inflammatory TME. Surprisingly, both the RNH1 high expression group and C1 group exhibited higher immune activation scores for the immune cycle. These findings establish for the first time a relationship between RNH1 and immunity. They also suggested that RNH1 plays an important role in immunotherapy.

Molecular subtypes can predict the response to immunotherapy, radiotherapy, neoadjuvant chemotherapy, and several targeted therapies^2,52^. We found that RNH1 expression was significantly associated with multiple BLCA-related signalling pathways. Notably, RNH1 expression was positively related to the enrichment scores of therapeutic signalling pathways. Furthermore, RNH1 expression predicted the response to therapeutic options in BLCA. In terms of drug response, high expression of RNH1 could make cancer cells more sensitive to cetuximab. There was a notably better response to ERBB therapy in the high-RNH1 group. This has important guiding significance for exploring the neoadjuvant combination therapy for BLCA^53^.

In conclusion, our study revealed that RNH1 affects multiple functional states of BLCA, especially EMT and invasion, and can predict BLCA invasion and metastasis. Moreover, we found that RNH1 expression is significantly associated with multiple therapeutic signalling pathways and drug targets in BLCA. We believe that RNH1 can provide new insights into the invasion and migration of BLCA and predict the treatment response in patients with BLCA.

### Supplementary Information

Supplementary Figures.

Supplementary Tables.

## Data Availability

All data generated or analyzed during this study are included in this article and its supplementary information files. These data are also available in the following databases (https://portal.gdc.cancer.gov/, https://www.ncbi.nlm.nih.gov/geo/, http://timer.cistrome.org/, http://gepia2.cancer-pku.cn/#analysis, http://ualcan.path.uab.edu/analysis-prot.html, https://go.drugbank.com/, https://www.cbioportal.org/, https://string-db.org/, http://biocc.hrbmu.edu.cn/CancerSEA/).
