# Supplementary material for: Functional status analysis of RNH1 in bladder cancer for predicting immunotherapy response
Source: Sci Rep. 2023 Aug 3;13:12625. doi: 10.1038/s41598-023-39827-7 (PMC10400633; doi:10.1038/s41598-023-39827-7)
Supplement: Supplementary file 1 — Supplementary Figures. [file 41598_2023_39827_MOESM1_ESM.docx]

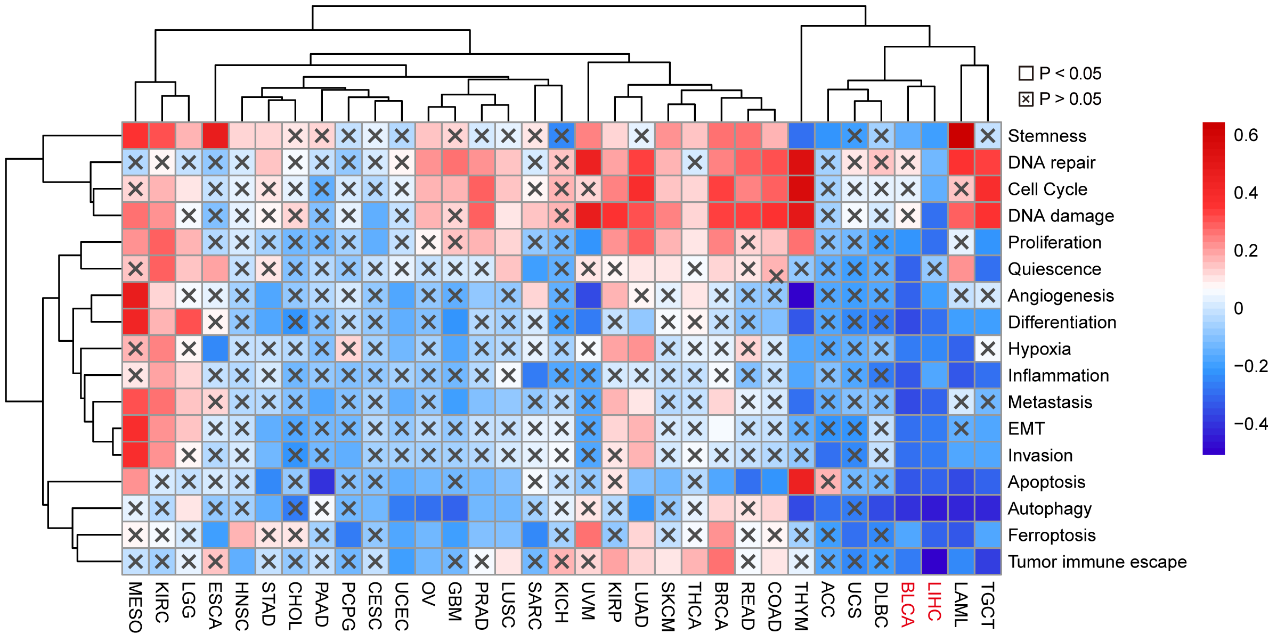


**Figure S1.** Heatmap of correlations between RNH1 expression and functional scores in 33 cancer types. × represents *p* > 0.05.


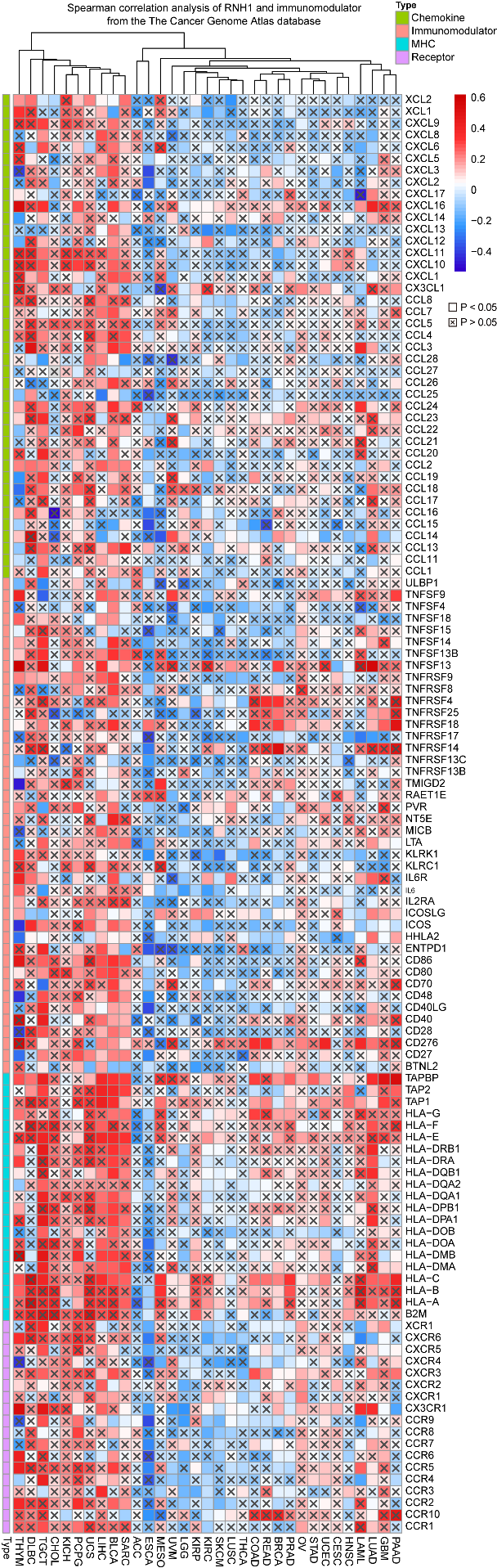


**Figure S2.** Spearman correlation heatmap showing the relationship between RNH1 expression and immunomodulators.


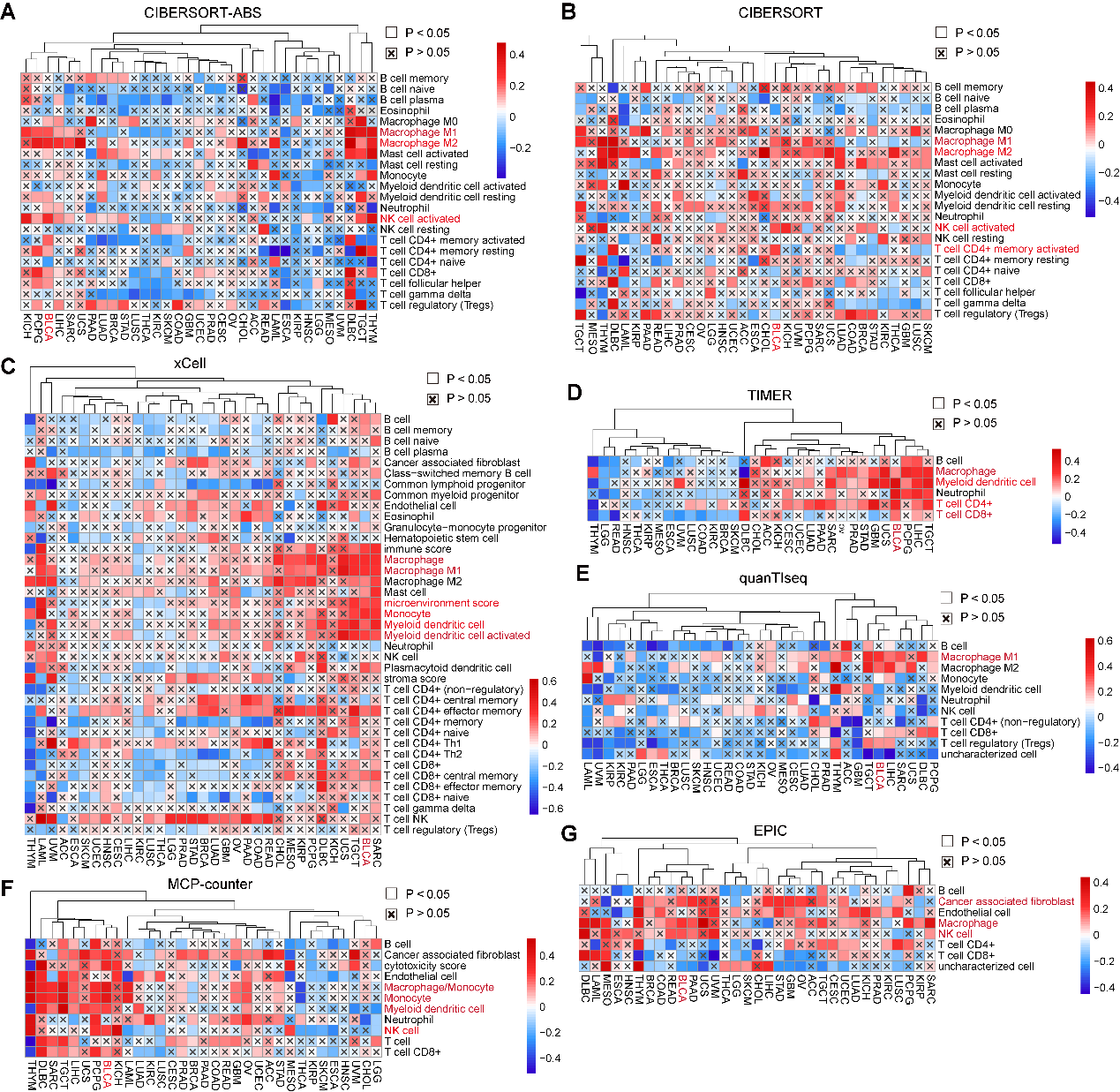


**Figure S3. Spearman correlation heatmap showing the relationship between *RNH1* expression and tumor infiltrating leukocytes (TILs) based on different algorithms. (A)** CIBERSORT-ABS, **(B)** CIBERSORT, **(C)** xCell, **(D)** TIMER, **(E)** quantTIseq, **(F)** MCP-counter, and **(G)** EPIC.
